# The Impact of Antithrombotic Therapy on Bleeding Complications in Percutaneous Liver Biopsy: Are the Withdrawal Criteria of Antithrombotic Agents in Japan Gastroenterological Endoscopy Society Guidelines for Gastroenterological Endoscopy Useful?

**DOI:** 10.3390/diseases14060184

**Published:** 2026-05-22

**Authors:** Kensuke Kitsugi, Yoshisuke Hosoda, Go Murohisa, Yashiro Yoshizawa, Masaharu Kimata, Yosuke Kobayashi, Shuhei Unno, Toshihiro Takayanagi, Kazuhito Kawata

**Affiliations:** 1 Department of Gastroenterology, Seirei Hamamatsu General Hospital, Shizuoka 430-8558, Japan; yhosoda@sis.seirei.or.jp (Y.H.); murohisa@sis.seirei.or.jp (G.M.); yashiro1224@sis.seirei.or.jp (Y.Y.); masa-kimata@sis.seirei.or.jp (M.K.); koba0706@msn.com (Y.K.); shuhei.sora@gmail.com (S.U.); aciel00009648@outlook.jp (T.T.); 2 Department of Internal Medicine II, Hamamatsu University School of Medicine, Shizuoka 431-3192, Japan; kawata@hama-med.ac.jp

**Keywords:** anticoagulant agents, antiplatelet agents, platelet counts, prothrombin time, serum albumin

## Abstract

Background: Bleeding is the most important complication of percutaneous liver biopsy. However, there are many unknowns regarding the management of antithrombotic agents in percutaneous liver biopsy. We investigated whether percutaneous liver biopsy could be performed safely by withdrawal of antithrombotic agents in accordance with the criteria of gastroenterological endoscopy guidelines. Methods: A retrospective study was conducted on patients who underwent percutaneous liver biopsy. Antithrombotic agents were discontinued in accordance with the withdrawal criteria in the Japan Gastroenterological Endoscopy Society guidelines for gastroenterological endoscopy. Results: A total of 630 cases were enrolled, including 64 cases receiving antithrombotic therapy. Bleeding complications occurred in 6.7% of cases with antithrombotic therapy and 2.1% of patients without antithrombotic therapy, with no significant difference between the two groups (*p* = 0.069). Major bleeding requiring surgical intervention or transarterial embolization was not observed in cases with antithrombotic therapy. Only one case (1.6%) developed thromboembolism after withdrawal of antithrombotic agents. Even after propensity score matching, there was no significant difference in the bleeding complication rates between the cases with and without antithrombotic therapy in either tumor biopsy (*p* = 0.599) and non-tumor biopsy (*p* = 0.440). Univariate and multivariate analysis revealed that low platelet count (≤10 × 10^4^/μL) was a significant risk factor for bleeding complications (OR = 1.07, 95%CI 1.01–1.15, *p* = 0.034), whereas antithrombotic therapy was not a significant factor (OR = 2.7, 95%CI 0.79–9.22, *p* = 0.114). Conclusions: This study suggests that percutaneous liver biopsy may be performed safely in cases receiving antithrombotic therapy by discontinuation of antithrombotic agents in accordance with the withdrawal criteria of Japan Gastroenterological Endoscopy Society guidelines for gastroenterological endoscopy.

## 1. Introduction

Percutaneous liver biopsy is an essential tool for the diagnosis and staging of liver disease and liver tumors [1]. With the remarkable development of personalized medicine, especially in the field of oncology, the importance of histological diagnosis by liver biopsy is increasing [2]. Biopsy with real-time ultrasonography imaging has improved the diagnostic accuracy and safety [3]. However, percutaneous liver biopsy still carries the risk of serious complications [4].

Bleeding is the most common and serious complication in percutaneous liver biopsy, occurring in up to 10.9% of cases [5]. The main source of mortality following liver biopsy is bleeding complications [6]. Hemoperitoneum, hemothorax, biliary tract hemorrhage, or subcapsular liver hematoma have been reported as bleeding complications in percutaneous liver biopsy, and major bleeding requiring additional treatments, such as interventional or surgical treatment, occurs in 0.1–4.6% of cases [7]. Risk factors for bleeding range from age and coagulation ability to tumor biopsy and number of punctures [7,8]. However, there are no guidelines regarding liver biopsy in Japan, and consensus regarding the risk factors for bleeding complications or the management of high-risk cases has not been established.

Antithrombotic therapy may also affect bleeding complications in percutaneous liver biopsy, but there is a lack of evidence regarding peribiopsy management in cases with antithrombotic therapy. The American Association for the Study of Liver Diseases (AASLD) position paper on liver biopsy suggests withdrawal of antithrombotic agents for several days to 10 days [3]. However, the statement notes that there is a lack of evidence regarding bleeding risk for patients receiving newer antiplatelet agents such as thienopyridine derivatives and direct oral anticoagulants (DOAC). Guidelines published by the British Society of Gastroenterology, the Royal College of Radiologists, and the Royal College of Pathology specify recommended withdrawal periods of antithrombotic agents, including thienopyridine derivatives and DOAC [9]. However, the guidelines recommend that withdrawal of antithrombotic agents should be considered carefully on an individual basis because of the lack of evidence. Moreover, these guidelines state that the appropriate timing for restarting antithrombotic agents after discontinuation is unclear.

In Japan, withdrawal of antithrombotic agents in gastrointestinal endoscopy is performed in accordance with the Japan Gastroenterological Endoscopy Society (JGES) guidelines for gastroenterological endoscopy [10,11]. A distinctive feature of these guidelines is that the withdrawal period is set with an emphasis on the risk of thromboembolism associated with discontinuing antithrombotic agents, rather than the risk of bleeding. Thromboembolism associated with withdrawal of antithrombotic agents can be serious, and long withdrawal periods may worsen patient outcomes [12,13]. Because of the global aging population, the number of patients taking antithrombotic agents is increasing. Therefore, it is necessary to consider not only the risk of bleeding but also the risk of thromboembolism when performing a percutaneous liver biopsy. Management of antithrombotic agents according to the JGES guidelines, focusing on the risk of thromboembolism, may also be useful in percutaneous liver biopsy. However, bleeding complications in liver biopsy are more difficult to control than those in gastrointestinal endoscopic procedures. Therefore, it is necessary to investigate whether the withdrawal period in the JGES guidelines is appropriate for percutaneous liver biopsy in cases with antithrombotic therapy.

In this study, we investigated whether percutaneous liver biopsy could be performed safely by discontinuation of antithrombotic agents in accordance with the withdrawal criteria of JGES guidelines for gastroenterological endoscopy.

## 2. Materials and Methods

### 2.1. Study Population

**Figure 1:**
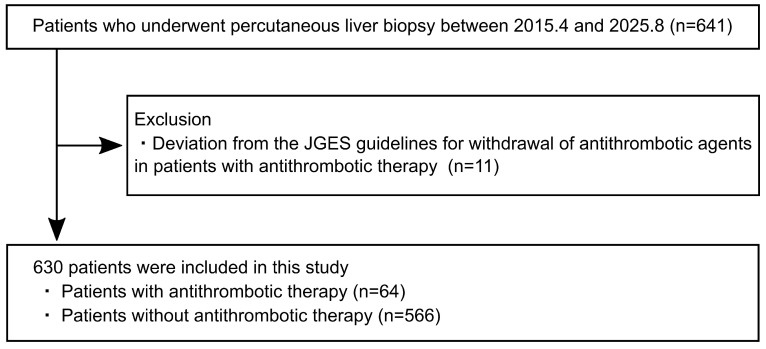
Flow diagram of the study. JGES, Japan Gastroenterological Endoscopy Society.

The study was a single-center retrospective observation. The study’s population consisted of consecutive patients who underwent percutaneous liver biopsy at Seirei Hamamatsu General Hospital between April 2015 and August 2025. For cases with antithrombotic therapy, the withdrawal period of antithrombotic agents was determined at the discretion of the attending physician. Patients who discontinued antithrombotic agents in accordance with the withdrawal criteria for high-risk procedures of bleeding in the JGES guidelines for gastroenterological endoscopy [10,11] were included in this study as the antithrombotic therapy group. The JGES guidelines stipulate a different withdrawal period of antithrombotic agents depending on the bleeding risk of the procedure. We defined the percutaneous liver biopsy as a high-risk bleeding procedure, similar to endoscopic submucosal dissection and endoscopic ultrasound-guided fine needle aspiration. Patients deviating from the guidelines were excluded (*n* = 11, 1.7%). The remaining 630 cases were enrolled in this study (Figure 1). This study was approved by the Ethics Committee of the Seirei Hamamatsu General Hospital (Ethics Approval Number: 4952). Each patient was offered the opportunity to decline participation in the study through an opt-out option.

### 2.2. Biopsy Procedures

All procedures were performed by experts or by trainees under their direct supervision, utilizing real-time ultrasonography. An automatic (Acecut ^®^; TSK Laboratory, Tochigi, Japan) or semi-automatic (TEMNO Evolution ^®^; Merit Medical Japan, Tokyo, Japan) biopsy needle was selected at the discretion of the operators. An 18-gauge biopsy needle was selected for liver tumor biopsies, and a 16-gauge biopsy needle was selected for non-tumor biopsies. One to four passes were performed to obtain sufficient tissue samples. After the procedure, the patients were placed in a supine position, and closely monitored for at least 24 h after the biopsy. All patients were monitored for delayed bleeding in an outpatient setting for at least 10 days. Computed tomography (CT) was performed for patients with signs and symptoms suggestive of bleeding complications.

### 2.3. Antithrombotic Agents

The antithrombotic agents were discontinued in accordance with the withdrawal criteria for high risk of bleeding in the JGES guidelines [10,11]. The specifics were as follows: (1) aspirin was continued or discontinued for 3–5 days depending on thromboembolic risk; (2) thienopyridine derivatives were discontinued for 5–7 days; (3) antiplatelet agents other than aspirin and thienopyridine were discontinued for one day; (4) warfarin was replaced with heparin; (5) DOAC were discontinued on the morning of the procedure; (6) In patients taking aspirin in combination with other antithrombotic agents, the procedures were carried out on monotherapy with aspirin and other antithrombotic agents were discontinued based on above criteria (Table 1). After the temporary withdrawal, the antithrombotic agents were resumed the day after the procedure. In cases of bleeding complications, the antithrombotic agents were resumed as soon as hemostasis had been confirmed.

### 2.4. Evaluations and Definitions

The clinical and demographic characteristics of patients and laboratory data were collected from medical records. The laboratory data were obtained on the day of liver biopsy. Bleeding complications were defined as any of the following events: (1) CT evaluation demonstrating hemoperitoneum, hemothorax, biliary tract hemorrhage, or subcapsular liver hematoma; (2) decrease in hemoglobin concentration of 2 g/dL or more after the procedures. Major bleedings were defined as those requiring additional treatments, such as interventional or surgical treatment. All bleeding complications in this study were observed within 24 h, and delayed bleeding complications were not observed.

### 2.5. Statistical Analyses

Statistical sample size calculations were not conducted due to the retrospective nature of the study. Post hoc power analysis revealed that the power to detect bleeding rates after percutaneous liver biopsy was 39%. Data on patient characteristics are presented as numbers for categorical data, and medians and total range for continuous variables. The Mann–Whitney U test was used to compare continuous variables. Categorical variables were compared using Fisher’s exact test. Univariate and multivariate analyses were performed using a logistic regression model for predicting the bleeding complications. The Cochrane–Armitage test was performed to evaluate the trend of binomial proportions. Propensity scores were estimated by a logistic regression model, with observed variables such as sex, age, type of liver biopsy (tumor biopsy or non-tumor biopsy), serum albumin level, estimated glomerular filtration rate (eGFR), and platelet count. A matching between cases with antithrombotic therapy and without antithrombotic therapy was performed according to the nearest neighbor method using a 0.2-width caliper and a 1:3 ratio. One-way analysis of variance followed by Bonferroni’s post hoc test to compare the means of three or more samples. All analyses were performed using EZR, a modified version of the R commander designed to add statistical functions frequently used in biostatistics [14]. A *p*-value of < 0.05 was considered statistically significant.

## 3. Results

### 3.1. Patient Characteristics

The patient characteristics are summarized in Table 2. The median age was 65 years. Males comprised 277 cases (44.0%), while females comprised 353 cases (56.0%). Liver tumor biopsy was performed in 306 cases (48.6%), while non-tumor liver biopsy was performed in 324 cases (51.4%). The total number of punctures was once in 372 cases (59.0%), twice in 178 cases (28.3%), three times in 54 cases (8.6%), and four times in 26 cases (4.1%). In the laboratory data, the median values of total bilirubin, aspartate aminotransferase (AST), alanine aminotransferase (ALT), alanine aminotransferase (ALP), gamma-glutamyl transferase (GGT), albumin, and eGFR, were 0.8 mg/dL, 48 U/L, 43 U/L, 132 U/L, 118 U/L, 3.8 g/dL, and 78 mL/min/1.73 m^2^, respectively. The median value of platelet count was 21.5 × 10^4^/μL, with 39 cases (6.2%) below 10.0 × 10^4^/μL and one case (0.2%) below 5.0 × 10^4^/μL. In the coagulation tests, activated partial thromboplastin time (APTT), prothrombin time-international normalized ratio (PT-INR), and fibrinogen levels were 30.8 s, 0.99, and 311 mg/dL, respectively. Sixty-four cases (10.2%) received antithrombotic therapy. Details of antithrombotic therapy are shown in Table S1. Fifty-four cases received monotherapy and 10 cases received dual therapy. The most common agent of monotherapy was DOAC with 12 cases, followed by aspirin with 11 cases, eicosapentaenoic acid (EPA) ethyl ester with 10 cases, warfarin with 8 cases, and thienopyridine with 7 cases. All dual therapy regimens were combined with aspirin. Concomitant use of aspirin included thienopyridine in 4 cases, warfarin in 3 cases, EPA ethyl ester in 2 cases, and DOAC in one case. Aspirin was continued in 9 cases and discontinued in 2 cases for 6 days in monotherapy. In dual therapy, aspirin was continued in all cases. The most common underlying diseases for antithrombotic therapy were ischemic heart disease and cerebrovascular disease with 15 cases, followed by atrial fibrillation with 12 cases, deep vein thrombosis with 6 cases, and arteriosclerosis obliterans (ASO) and after valvular surgery with 2 cases. In addition to diseases with a risk of thromboembolism, EPA ethyl ester was administered for hyperlipidemia and limaprost for lumbar spinal stenosis.

In a comparison between cases with antithrombotic therapy and without antithrombotic therapy, there were significant differences in age, the proportion of gender and tumor biopsy, renal function, and the values of serum albumin, APTT, PT-INR, and fibrinogen.

### 3.2. Bleeding Complications and Thromboembolism

**Figure 2:**
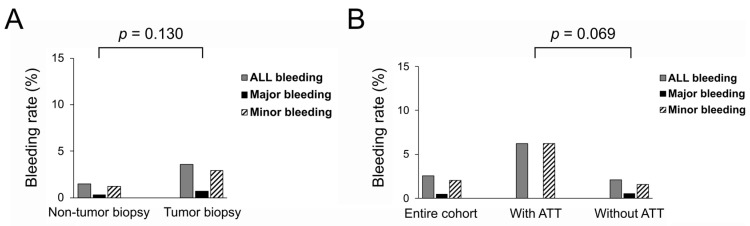
The impact of types of liver biopsy and antithrombotic therapy on bleeding complications. Bleeding complication rates in tumor biopsy and non-tumor biopsy (**A**), and bleeding complication rates with antithrombotic therapy and without antithrombotic therapy (**B**). ATT, antithrombotic therapy.

Details of bleeding complications are summarized in Table 3. Bleeding complications were observed in 16 cases (2.5%). Among them, 3 cases (0.5%) had major bleeding and 13 cases (2.0%) had minor bleeding. Regarding the type of bleeding complication, 11 cases (1.7%) were hemoperitoneum, 3 cases (0.5%) were hemothorax, and 2 cases (0.3%) were biliary tract hemorrhage. In cases with major bleeding, 2 cases underwent surgical intervention and one case underwent transarterial embolization. In all other cases, hemostasis was achieved with conservative treatment. Considering the influence of time period bias by improving peribiopsy management, the observation period was divided into two equal parts, but no significant difference in bleeding rates was observed depending on the time period (early period vs. late period, 3.1 vs. 9.4%, *p* = 0.613). Bleeding complications by type of liver biopsy are shown in Figure 2A. The bleeding rates for tumor and non-tumor biopsies were 3.6% and 1.5%, major bleeding 0.7% and 0.3%, and minor bleeding 2.9% and 1.2%, respectively. There was no significant difference in the incidence of bleeding complications between tumor and non-tumor biopsies (*p* = 0.130). In cases with antithrombotic therapy, bleeding complications were observed in 4 cases (6.3%), all of which were minor bleeding. All bleeding complications in antithrombotic therapy were cases with monotherapy, and bleeding complications were not observed in cases with dual therapy. In cases without antithrombotic therapy, 12 cases (2.1%) had bleeding complications, including 3 major bleeding (0.5%) and 9 minor bleeding (1.6%). There was no significant difference in the incidence of bleeding complications between cases with antithrombotic therapy and without antithrombotic therapy (*p* = 0.069) (Figure 2B). Thromboembolism associated with withdrawal of antithrombotic agents was observed in only one case (1.6%), receiving sarpogrelate for ASO. The patient experienced the deterioration of ASO the day after liver biopsy and improved with surgical intervention.

### 3.3. Characteristics in Cases with Antithrombotic Therapy and Without Antithrombotic Therapy in Non-Tumor Biopsy Before and After Propensity Score Matching

We next investigated the impact of antithrombotic therapy on bleeding complications in a non-tumor biopsy. We adjusted patient characteristics in cases with antithrombotic therapy and without antithrombotic therapy using propensity score matching. In addition to age, gender, and number of punctures, we adjusted for patient background factors that have been reported to be associated with bleeding complications in percutaneous liver biopsy, including platelet count, serum albumin level, and renal function [15,16,17,18]. Coagulation activity, such as APTT, PT-INR, and plasma fibrinogen activity, may also affect bleeding complications [15]. However, considering that antithrombotic agents affect coagulation activity [19], we did not adjust these parameters. Patient characteristics before and after matching are summarized in Table 4. Before matching, there were significant differences in age and the proportion of gender. After matching, the study cohort comprised 18 cases with antithrombotic therapy and 54 cases without antithrombotic therapy. No significant differences were observed between the two groups in any of the variables. There was no significant difference in the incidence of bleeding complications (*p* = 0.440).

### 3.4. Characteristics of Cases with and Without Antithrombotic Therapy in Tumor Biopsy Before and After Propensity Score Matching

We next investigated the impact of antithrombotic therapy on bleeding complications in tumor biopsy. As with the investigation in non-tumor biopsy, we adjusted patient characteristics using propensity score matching. Patient characteristics before and after matching are summarized in Table 5. Before matching, there were significant differences in age, the proportion of gender, serum albumin levels, renal function, and coagulation activity, suggesting that cases with antithrombotic therapy had a higher risk of bleeding. After matching, the study cohort comprised 35 cases with antithrombotic therapy and 105 cases without antithrombotic therapy. Only the values of PT-INR were significantly higher in cases with antithrombotic therapy. As with the results of non-tumor biopsy, there was no significant difference in the incidence of bleeding complications (*p* = 0.599).

### 3.5. The Investigation of the Factors Associated with Bleeding Complications in Percutaneous Liver Biopsy

**Figure 3:**
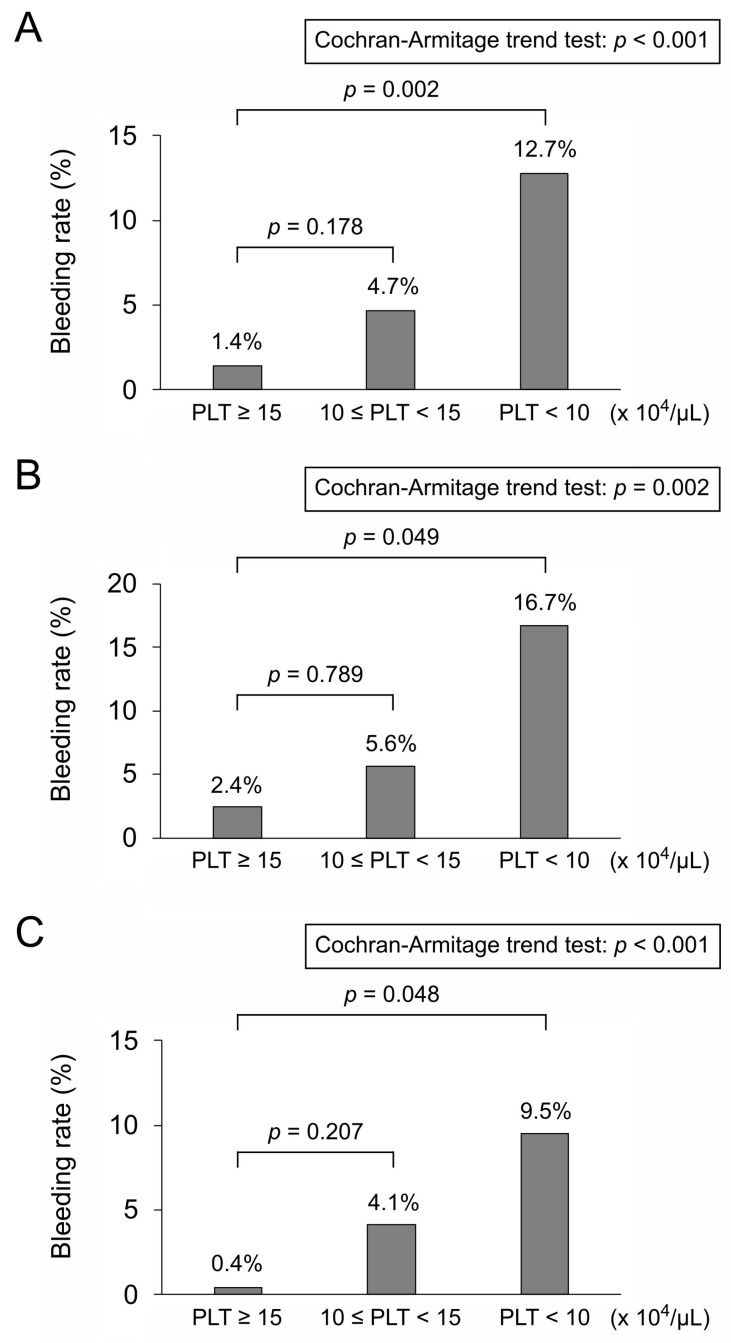
The incidence of bleeding complications classified by platelet count. Correlation between platelet count and bleeding complications in the entire cohort (**A**), tumor biopsy (**B**), and non-tumor biopsy (**C**). Fisher’s exact tests with Bonferroni correction were performed. PLT, platelet count.

We next performed univariate and multivariate analyses in the entire cohort to identify the predictive factors for bleeding complications in percutaneous liver biopsy (Table 6). Univariate analysis revealed that the serum albumin level (*p* = 0.023), platelet count (*p* = 0.031), and PT-INR (*p* = 0.009) were significantly associated with the bleeding complications. Multivariate analysis revealed that platelet count (*p* = 0.034) was independently associated with the bleeding complications. Antithrombotic therapy was not a significant factor in either univariate or multivariate analyses. In the entire cohort, the Cochrane–Armitage trend test revealed that the bleeding complication rate increased with decreasing platelet count (*p* < 0.001). Moreover, a significant increase in bleeding complications was observed when the platelet count was ≤10 × 10^4^/μL (*p* = 0.002) (Figure 3A). These results were consistent across cases with tumor biopsy and non-tumor biopsy (Figure 3B,C). Subgroup analysis of cases with antithrombotic therapy revealed no significant factors associated with bleeding complications, including platelet count (Table S2). However, there were only two cases with platelet count below 10 × 10^4^/μL in cases with antithrombotic therapy.

## 4. Discussion

In this study, we investigated the impact of antithrombotic therapy on bleeding complications in percutaneous liver biopsy. This study suggests that percutaneous liver biopsy may be performed safely in cases with antithrombotic therapy by managing antithrombotic agents in accordance with the withdrawal criteria of the JGES guidelines for gastroenterological endoscopy. Notably, no patients experienced major bleeding or fatal thromboembolism events in the antithrombotic therapy group. As shown in Figure 2, the bleeding complication rate was higher in cases with antithrombotic therapy compared to cases without antithrombotic therapy. However, a significant difference was not observed, and antithrombotic therapy was not a significant predictor of bleeding complications in univariate and multivariate analysis. Moreover, as shown in Table 2, cases with antithrombotic therapy were likely at a higher risk of bleeding due to factors such as a higher proportion of tumor biopsy and older age. A key finding of this study is that antithrombotic therapy did not significantly increase the bleeding complications upon discontinuation of antithrombotic agents in accordance with JGES guidelines, despite the cases with antithrombotic therapy having a higher risk of bleeding.

In Japan, there are no guidelines regarding liver biopsies, and the handling of antithrombotic agents has been left to the discretion of each institution. In this study, withdrawal of antithrombotic agents was performed in accordance with the withdrawal criteria for high-risk procedures of bleeding in the JGES guidelines [10,11]. This guideline recommends shorter withdrawal periods for most antithrombotic agents than foreign guidelines [3,10,11]. This is based on the fact that thromboembolism due to withdrawal of antithrombotic agents can be serious and potentially fatal. There is concern that shortening the withdrawal period may lead to an increased risk of bleeding complications, but this study did not demonstrate a significant increase in bleeding complications. Because bleeding complications associated with liver biopsy are often more difficult to control than those associated with endoscopic procedures, attempts to shorten the withdrawal period of antithrombotic agents have been rarely investigated. The number of patients taking antithrombotic agents is on the rise due to the increase in coronary artery and cerebrovascular diseases caused by the global aging population [20,21]. We believe that this study will provide new insights into the management of antithrombotic agents in percutaneous liver biopsy.

There is a lack of evidence regarding the impact of antithrombotic therapy on percutaneous liver biopsy. Among the antithrombotic agents, aspirin has been the most widely investigated. According to British guidelines, percutaneous liver biopsy can be performed safely after a three-day aspirin withdrawal period, and biopsy can be performed while taking aspirin in emergency cases [9]. This recommendation is consistent with the JGES guidelines. A review by Atwell et al. demonstrated that aspirin use within 10 days did not increase bleeding complications [15]. On the other hand, a review by Potretzke et al. demonstrated that aspirin use within three days was significantly associated with bleeding complications [22]. In our study, in accordance with the JGES guidelines, aspirin was continued in most cases of monotherapy (82%) and in all cases of dual therapy. As a result, only one case of minor bleeding was observed. A previous study demonstrated that platelet hemostatic function can recover in most cases within three days of aspirin withdrawal [23]. In this study, no patients underwent platelet function tests. Platelet dysfunction induced by aspirin may also be a contributing factor to bleeding complications [24]. The only bleeding case in a patient taking aspirin in this study had a platelet count of over 10 × 10^4^/μL, suggesting that impaired platelet function may have been involved in bleeding complications. In cardiac surgery, a preoperative platelet function test was useful in predicting the risk of postoperative bleeding in cases with antiplatelet therapy [25]. Previous reports and our findings suggest that percutaneous liver biopsy may be performed safely under continuous or short-term aspirin withdrawal, but further investigation is required to evaluate the impact of platelet function on bleeding complications in percutaneous liver biopsy.

The use of thienopyridine derivatives is increasing in the treatment of coronary artery disease, but their impact on percutaneous liver biopsy has not been well investigated. The AASLD states that there is insufficient evidence to make recommendations [3]. British guidelines recommend a 7-day drug withdrawal, but note that the hemostatic effect may be difficult to reverse [9]. The JGES guidelines recommend a five-day withdrawal period for thienopyridine derivatives, based on the fact that there have been no reports of an increase in bleeding complications [10]. In our study, no bleeding complications or thromboembolic events were observed with a 5–7-day withdrawal period for thienopyridine derivatives in either monotherapy or dual therapy. There is a critical lack of evidence for antiplatelet agents other than aspirin and thienopyridine derivatives. The JGES guidelines state that a one-day withdrawal is sufficient for other antiplatelet agents [10]. However, we experienced bleeding complications in patients taking EPA ethyl ester and limaprost. Although EPA ethyl ester has a long half-life, it is not known to increase the risk of bleeding [26]. Limaprost is known to increase the risk of bleeding, especially when used in combination with other antiplatelet agents [27]. In our case, limaprost was administered as a monotherapy. Therefore, it is possible that EPA ethyl ester and limaprost may have little influence on the bleeding complications in our cases. Regarding antiplatelet agents other than aspirin, there is a lack of evidence, and further investigations are required.

Regarding warfarin, both the AASLD and British guidelines recommend a 5-day withdrawal and heparin replacement on an as-needed basis, which is the same as the JGES guidelines [3,9,10]. The JGES guidelines state that patients taking warfarin are at high risk of thromboembolism [10]. We also consulted with the prescribing doctors and performed heparin replacement, and neither bleeding complications nor thromboembolism was observed. However, some reports have demonstrated that heparin replacement has a higher bleeding rate than continuation of warfarin in endoscopic procedures [28]. Further evidence is required regarding warfarin management in percutaneous liver biopsy. British guidelines recommend a 2-day DOAC withdrawal [9]. The JGES guidelines recommend withdrawal of DOAC only on the day of endoscopic procedures [11]. Patients taking DOAC can reduce the risk of bleeding complications by avoiding the time of peak blood concentration during procedures [29]. Therefore, the procedure can be performed during the trough concentration period by withdrawal of DOAC on the day of procedure, and the risk of thromboembolism can be reduced by resuming the DOAC the day after the procedure [11]. In our study, minor bleeding was observed in only one case (8.3%) by withdrawal of DOAC on the day of the procedure, and thromboembolism was not observed.

Another important finding of this study was that resuming antithrombotic agents the day after the procedure was safe. There is little evidence regarding the timing of resumption of antithrombotic agents in percutaneous liver biopsy. The AASLD states that there is little evidence available regarding resumption of antithrombotic agents [3], and British guidelines do not make any statements [9]. Most bleeding complications in percutaneous liver biopsy are observed within a few hours of the procedure, but there have also been reports of delayed bleeding [30,31]. In this study, patients were monitored for at least 10 days after percutaneous liver biopsy in outpatient settings, but delayed bleeding was not observed. The reported cases of delayed bleeding occurred 4 and 5 days after liver biopsy, respectively [30,31]. These results suggest that monitoring for delayed bleeding may be sufficient for about a week. However, considering that both reported cases of delayed bleeding originated from pseudoaneurysms [30,31], the possibility of bleeding complications occurring at a longer time cannot be ruled out. The JGES guidelines recommend that prompt resumption of antithrombotic agents after hemostasis is confirmed, and antithrombotic agents are resumed the day after endoscopic procedures in most cases [10]. In our study, antithrombotic agents were resumed the day after the procedure in all non-bleeding cases, and delayed bleeding was not observed. Further investigations are required to determine the appropriate timing for resuming antithrombotic agents in cases of bleeding.

The only predictor of bleeding complications in percutaneous liver biopsy in this study was thrombocytopenia. Various risk factors associated with bleeding complications in percutaneous liver biopsy have been investigated, and abnormalities in the blood coagulation system have been widely reported [7,15,16,17,18]. In particular, thrombocytopenia has been reported to be associated with bleeding complications in percutaneous liver biopsy [15,17,32]. It has been reported that patients with platelet counts of 6.0 × 10^4^/μL or higher in percutaneous liver biopsy do not have an increased risk of bleeding complications [33], and the AASLD also defines platelet counts of 6.0 × 10^4^/μL as the minimum safety threshold [3]. In our study, bleeding complications were significantly increased at platelet counts below 10.0 × 10^4^/μL, and the bleeding rate increased at higher thresholds compared with these reports. Previous studies have also demonstrated that platelet counts associated with bleeding complications range from 7.0 × 10^4^ to 19.0 × 10^4^/μL, which is a wide range of thresholds [15,16,34,35]. Platelet count does not reflect platelet function, and higher platelet counts may be required for hemostatic function in percutaneous liver biopsy. Transjugular liver biopsy may be an option in patients with thrombocytopenia [36]. Furthermore, univariate analysis demonstrated that PT-INR and serum albumin levels were predictive factors for bleeding complications. It has been reported that a high PT-INR alone is not a risk factor for bleeding [9]. Although PT-INR greater than 1.5 increases the risk of bleeding [3,9], the incidence of INR > 1.5 in this study was only 1.4%. Hypoalbuminemia has been reported to be associated with bleeding complications in percutaneous liver biopsy [16]. However, hypoalbuminemia is associated with bleeding complications but also with thromboembolism [37]. There are many unknowns regarding the relationship between coagulation activity, hypoalbuminemia, and bleeding complications in percutaneous liver biopsy.

Subgroup analysis in the antithrombotic therapy group revealed no factors associated with bleeding complications. We believe this is because the fact that the number of bleeding cases was very small in cases with antithrombotic therapy. Thrombocytopenia was also not significant in the antithrombotic therapy group. However, there were only two cases (3.1%) in the antithrombotic therapy group with platelet counts below 10.0 × 10^4^/μL. As shown in Table 2, the antithrombotic therapy group was significantly older, had a higher number of tumor biopsies and lower serum albumin levels and coagulation activity than the group without antithrombotic therapy. Therefore, we do not believe that the antithrombotic therapy group was necessarily at low risk of bleeding. Future investigation is desirable to evaluate the impact of platelet count in cases with antithrombotic therapy.

This study has some limitations. First, the number of cases was limited because this was a single-center retrospective study. Especially, the number of cases with antithrombotic therapy was also insufficient to enhance the robustness of the results. Furthermore, it was difficult to evaluate the risk factors for bleeding complications in the antithrombotic therapy group because the number of bleeding cases was limited. This also made it difficult to investigate the impact of antithrombotic therapy on different types of bleeding complications. Moreover, there is no standardization of procedures, such as the number of punctures or the selection of a biopsy needle. Therefore, it is necessary to conduct prospective studies with a larger number of cases at multiple institutions. Second, all cases in combination therapies of antithrombotic agents were dual therapy, and all involved the combination of aspirin with another antithrombotic agent. The appropriate strategies for triple therapy or combination therapy that does not include aspirin are unknown. Third, 11 cases (1.7%) were excluded because they deviated from the withdrawal criteria of the JGES guidelines. In these cases, antithrombotic agents had been discontinued for a longer period than the withdrawal criteria of the JGES guidelines. These cases were likely at a higher risk of bleeding, and the possibility of selection bias cannot be ruled out.

## 5. Conclusions

It may be possible to safely perform percutaneous liver biopsy even in cases receiving antithrombotic therapy by following the withdrawal criteria in the JGES guidelines for gastroenterological endoscopy. However, there is a possibility of an increased risk of bleeding complications in cases with low platelet counts. Therefore, appropriate patient selection is important.

## Figures and Tables

**Table 1:** Withdrawal of antithrombotic agents in this study.

Antithrombotic Agents	Duration of Withdrawal
Aspirin Thienopyridine Antiplatelet agents other than aspirin and thienopyridine Warfarin Direct oral anticoagulants	Continue or withdraw for 3–5 days Withdraw for 5–7 days Withdraw for one day Heparin replacement Withdraw on the day of the procedure
Dual therapy	Continue aspirin, and withdraw other antithrombotic agents according to the above criteria

**Table 2:** Patient characteristics.

Variable	Entire Cohort	with ATT	Without ATT	*p*-Value
	(n = 630)	(n = 64)	(n = 566)	
Age, median years (range)	65 (17–94)	73 (45–94)	63 (17–94)	<0.001
Male gender, n (%)	277 (44.0)	43 (67.2)	234 (41.3)	<0.001
Tumor biopsy, n (%)	306 (48.6)	44 (68.8)	262 (46.3)	0.001
Total number of punctures, n (%)				0.390
Once	372 (59.0)	34 (53.1)	338 (59.7)	
Twice	178 (28.3)	22 (34.4)	156 (27.6)	
Three times	54 (8.6)	5 (7.8)	49 (8.7)	
Four times	26 (4.1)	3 (4.7)	23 (4.0)	
Laboratory data, median (range)				
Total bilirubin [mg/dL]	0.8 (0.1–29.1)	0.8 (0.2–11.4)	0.8 (0.1–29.1)	0.375
AST [U/L]	48 (10–2446)	49 (13–2446)	48 (10–2177)	0.549
ALT [U/L]	43 (4–2972)	37 (7–2712)	44 (4–2972)	0.124
ALP [U/L]	132 (33–1756)	129 (39–1756)	133 (33–1529)	0.592
GGT [U/L]	118 (10–2711)	140 (17–1887)	117 (10–2711)	0.406
Albumin [g/dL]	3.8 (1.6–5.1)	3.6 (2.0–4.7)	3.8 (1.6–5.1)	0.004
eGFR [mL/min/1.73 m^2^]	78 (4–162)	71 (18–132)	79 (4–162)	0.001
Platelet count [×10^4^/μL]	21.5 (4.9–67.4)	21.4 (6.0–67.4)	21.6 (4.9–65.3)	0.583
APTT [s]	30.8 (19.1–76.6)	33.1 (22.0–76.6)	30.1 (19.1–67.1)	0.027
PT-INR	0.99 (0.72–1.84)	1.04 (0.73–1.84)	0.98 (0.72–1.70)	<0.001
Fibrinogen [mg/dL]	311 (96–800)	341 (110–680)	310 (96–800)	0.041

AST, aspartate aminotransferase; ALT, alanine aminotransferase; ALP, alkaline phosphatase; GGT, gamma-glutamyl transferase; eGFR, estimated glomerular filtration rate; APTT, activated partial thromboplastin time; PT-INR, prothrombin time-international normalized ratio ATT; antithrombotic therapy.

**Table 3:** Details of bleeding complications.

Variable	Entire Cohort	with ATT	Without ATT
	(n = 630)	(n = 64)	(n = 566)
All bleeding complications, n (%)	16 (2.5)	4 (6.3)	12 (2.1)
Severity and type of bleeding complications, n (%)			
Major bleeding			
Hemoperitoneum	2 (0.3)	0 (0)	2 (0.3)
Hemothorax	1 (0.2)	0 (0)	1 (0.2)
Biliary tract hemorrhage	0 (0)	0 (0)	0 (0)
Minor bleeding			
Hemoperitoneum	9 (1.4)	3 (4.7)	6 (1.1)
Hemothorax	2 (0.3)	0 (0)	2 (0.3)
Biliary tract hemorrhage	2 (0.3)	1 (1.6)	1 (0.2)
Procedures for bleeding complications, n (%)			
Surgical intervention	2 (0.3)	0 (0)	2 (0.3)
Transarterial embolization	1 (0.2)	0 (0)	1 (0.2)
Conservative treatment	13 (2.0)	4 (6.3)	9 (1.6)

ATT, antithrombotic therapy.

**Table 4:** Comparison of the patient characteristics in cases with antithrombotic therapy and without antithrombotic therapy in non-tumor biopsy.

	Before Matching			After Matching		
Variable	with ATT	Without ATT	*p*-Value	with ATT	Without ATT	*p*-Value
	(n = 20)	(n = 304)		(n = 18)	(n = 54)	
Age, median years (range)	70 (45–82)	61 (17–87)	0.002	69 (45–81)	67 (45–87)	0.416
Male gender, n (%)	12 (60.0)	101 (33.2)	0.027	10 (55.6)	31 (57.4)	1.000
Number of punctures, median (range)	1 (1–2)	1 (1–4)	0.794	1 (1–2)	1 (1–3)	0.302
Laboratory data, median (range)						
Total bilirubin [mg/dL]	1.1 (0.5–11.4)	0.9 (1.0–4.0)	0.368	1.1 (0.5–11.4)	1.0 (0.3–18.0)	0.658
AST [U/L]	109 (18–2446)	85 (10–2177)	0.805	109 (18–2446)	97 (11–1753)	0.933
ALT [U/L]	98 (27–2712)	102 (4–2972)	0.615	98 (29–2712)	98 (9–2972)	0.558
ALP [U/L]	144 (49–1756)	149 (33–1529)	0.759	144 (49–287)	152 (62–1145)	0.594
GGT [U/L]	215 (23–1887)	153 (11–1834)	0.403	215 (23–598)	200 (12–1834)	0.785
Albumin [g/dL]	3.6 (2.2–4.5)	3.8 (1.6–5.1)	0.506	3.8 (2.2–4.5)	3.8 (2.2–4.5)	0.912
eGFR [mL/min/1.73 m^2^]	73 (50–130)	81 (6–162)	0.121	73 (50–118)	71 (6–123)	0.990
Platelet count [×10^4^/μL]	20.6 (7.9–34.2)	20.5 (5.3–53.9)	0.657	19.6 (7.9–34.2)	20.6 (5.3–39.5)	0.907
APTT [s]	32.2 (23.3–58.6)	32.1 (20.0–67.1)	0.558	32.2 (23.4–51.0)	32.0 (23.3–54.8)	0.677
PT-INR	1.02 (0.73–1.74)	0.98 (0.75–1.60)	0.587	1.02 (0.73–1.74)	0.99 (0.82–1.30)	0.691
Fibrinogen [mg/dL]	278 (197–659)	284 (96–800)	0.886	268 (197–659)	280 (156–566)	0.984
Breeding complications, n (%)	1 (5.0)	4 (1.3)	0.274	1 (5.6)	1 (1.9)	0.440

AST, aspartate aminotransferase; ALT, alanine aminotransferase; ALP, alkaline phosphatase; GGT, gamma-glutamyl transferase; eGFR, estimated glomerular filtration rate; APTT, activated partial thromboplastin time; PT-INR, prothrombin time-international normalized ratio; ATT, antithrombotic therapy.

**Table 5:** Comparison of the patient characteristics in cases with antithrombotic therapy and without antithrombotic therapy in tumor biopsy.

	Before Matching			After Matching		
Variable	with ATT	Without ATT	*p*-Value	with ATT	Without ATT	*p*-Value
	(n = 44)	(n = 262)		(n = 35)	(n = 105)	
Age, median years (range)	75 (52–94)	67 (21–94)	<0.001	73 (52–83)	74 (44–88)	0.711
Male gender, n (%)	31 (70.5)	133 (50.8)	0.021	24 (68.6)	64 (61.0)	0.545
Total number of punctures, median (range)	2 (1–4)	2 (1–4)	0.147	2 (1–4)	2 (1–4)	0.520
Laboratory data, median (range)						
Total bilirubin [mg/dL]	0.7 (0.2–5.4)	0.7 (0.1–16.9)	0.596	0.7 (0.3–5.4)	0.7 (0.1–16.9)	0.963
AST [U/L]	33 (13–198)	28 (10–423)	0.105	33 (13–149)	28 (13–366)	0.348
ALT [U/L]	23 (7–162)	24 (7–386)	0.966	22 (8–162)	24 (7–386)	0.870
ALP [U/L]	123 (39–713)	110 (40–947)	0.158	118 (39–713)	131 (40–851)	0.704
GGT [U/L]	123 (17–650)	66 (10–2711)	0.085	77 (17–650)	95 (12–987)	0.681
Albumin [g/dL]	3.6 (2.0–4.7)	3.8 (1.7–4.8)	0.003	3.7 (2.2–4.7)	3.6 (1.7–4.7)	0.896
eGFR [mL/min/1.73 m^2^]	64 (18–132)	76 (4–151)	0.013	72 (18–132)	71 (10–118)	0.782
Platelet count [×10^4^/μL]	21.4 (6.0–67.4)	22.3 (4.9–65.3)	0.967	21.1 (6.0–50.1)	21.9 (6.2–65.3)	0.590
APTT [s]	33.7 (22.0–76.6)	29.3 (19.1–66.3)	0.003	31.3 (25.0–76.6)	29.7 (23.2–66.3)	0.129
PT-INR	1.09 (0.88–1.84)	0.98 (0.72–1.70)	<0.001	1.08 (0.88–1.78)	0.99 (0.85–1.70)	<0.001
Fibrinogen [mg/dL]	393 (110–680)	351 (107–800)	0.157	388 (110–661)	407 (196–800)	0.431
Breeding complications, n (%)	3 (6.8)	8 (3.1)	0.200	2 (5.7)	3 (2.9)	0.599

AST, aspartate aminotransferase; ALT, alanine aminotransferase; ALP, alkaline phosphatase; GGT, gamma-glutamyl transferase; eGFR, estimated glomerular filtration rate; APTT, activated partial thromboplastin time; PT-INR, prothrombin time-international normalized ratio; ATT, antithrombotic therapy.

**Table 6:** Univariate and multivariate analysis for the bleeding complications in patient characteristics.

Variable	Univariate Analysis			Multivariate Analysis		
	OR	95% CI	*p*-Value	OR	95% CI	*p*-Value
Age	1.01	0.97–1.04	0.748			
Male gender	1.66	0.61–4.51	0.321			
Tumor biopsy	2.38	0.82–6.93	0.112			
Total number of punctures	1.22	0.74–2.03	0.437			
Laboratory data						
Total bilirubin	0.98	0.82–1.17	0.852			
AST	1.00	0.99–1.00	0.426			
ALT	1.00	0.99–1.00	0.235			
ALP	1.00	0.99–1.00	0.545			
GGT	1.00	0.99–1.00	0.839			
Albumin	2.23	1.12–4.47	0.023	2.08	0.92–4.73	0.079
eGFR	1.00	0.98–1.03	0.823			
Platelet count	1.08	1.01–1.15	0.031	1.07	1.01–1.15	0.034
APTT	1.00	0.93–1.07	0.974			
PT-INR	22.6	2.20–232.00	0.009	2.82	0.16–51.30	0.484
Fibrinogen	1.00	0.99–1.00	0.673			
Antithrombotic therapy	3.08	0.96–9.8	0.580	2.70	0.79–9.22	0.114

AST, aspartate aminotransferase; ALT, alanine aminotransferase; ALP, alkaline phosphatase; GGT, gamma-glutamyl transferase; eGFR, estimated glomerular filtration rate; APTT, activated partial thromboplastin time; PT-INR, prothrombin time-international normalized ratio.

## Data Availability

The data that support the findings of this study are available from the corresponding author upon reasonable request.

## Supplementary Materials

The following supporting information can be downloaded at: https://www.mdpi.com/article/10.3390/diseases14060184/s1, Table S1: Details of antithrombotic therapy (n = 64); Table S2: Univariate analysis for the bleeding complications in cases with antithrombotic therapy.

## Abbreviations

The following abbreviations are used in this manuscript:

AASLD	American Association for the Study of Liver Diseases
ALP	alanine aminotransferase
ALT	alanine aminotransferase
APTT	activated partial thromboplastin time
ASO	arteriosclerosis obliterans
AST	aspartate aminotransferase
ATT	Antithrombotic therapy
CT	computed tomography
DOAC	direct oral anticoagulants
eGFR	estimated glomerular filtration rate
EPA	eicosapentaenoic acid
GGT	gamma-glutamyl transferase
JGES	Japan Gastroenterological Endoscopy Society
PT-INR	prothrombin time-international normalized ratio
