# Supplementary material for: The Impact of Antithrombotic Therapy on Bleeding Complications in Percutaneous Liver Biopsy: Are the Withdrawal Criteria of Antithrombotic Agents in Japan Gastroenterological Endoscopy Society Guidelines for Gastroenterological Endoscopy Useful?
Source: Diseases. 2026 May 22;14(6):184. doi: 10.3390/diseases14060184 (PMC13298797; doi:10.3390/diseases14060184)
Supplement: Supplementary file 1 [file diseases-14-00184-s001.zip › diseases-4296864-supplementary.pdf]

Table S1.

Details of antithrombotic therapy (n=64)

**Variable**	**Results**
Antithrombotic therapy, n (%)	
Monotherapy	54 (84)
Aspirin	11 (17)
Thienopyridine	7 (11)
Cilostazol	1 (1)
Eicosapentaenoic acid ethyl ester	10 (16)
Limaprost	4 (6)
Sarpogrelate	1 (1)
Direct oral anticoagulants	12 (19)
Warfarin	8 (13)
Dual therapy	10 (16)
Aspirin plus thienopyridine	4 (6)
Aspirin plus warfarin	3 (5)
Aspirin plus eicosapentaenoic acid ethyl ester	2 (4)
Aspirin plus direct oral anticoagulants	1 (1)
Underlying disease for antithrombotic therapy, n (%)	
Ischemic heart disease	15 (23)
Cerebrovascular disease	15 (23)
Atrial fibrillation	12 (19)
Deep vein thrombosis	6 (9)
Arteriosclerosis obliterans	2 (4)
After valvular surgery	2 (4)
Hyperlipidemia	8 (12)
Lumbar spinal stenosis	4 (6)

Table S2.

Univariate analysis for the bleeding complications in cases with antithrombotic therapy

**Variable**	**Univariate analysis**		
	**OR**	**95% CI**	***p*-value**
Age	0.98	0.88-1.09	0.736
Male gender	1.50	0.15-15.40	0.733
Tumor biopsy	1.39	0.14-14.30	0.781
Total number of punctures	1.18	0.37-3.74	0.783
Laboratory data			
Total bilirubin	1.14	0.78-1.67	0.484
AST	1.00	0.99-1.00	0.838
ALT	1.00	0.99-1.01	0.738
ALP	1.00	0.99-1.00	0.226
GGT	1.00	0.99-1.00	0.138
Albumin	0.39	0.08-1.82	0.231
eGFR	1.00	0.99-1.00	0.838
Platelet count	1.00	0.90-1.10	0.924
APTT	0.96	0.84-1.09	0.536
PT-INR	1.90	0.03-126.00	0.764
Fibrinogen	1.00	0.99-1.01	0.733

Abbreviations: AST, aspartate aminotransferase; ALT, alanine aminotransferase; ALP, alkaline phosphatase; GGT, gamma-glutamyl transferase; eGFR, estimated glomerular filtration rate; APTT, activated partial thromboplastin time; PT-INR, prothrombin time-international normalized ratio.
